# Gluten-Free Sweet Potato Flour: Effect of Drying Method and Variety on the Quality and Bioactivity

**DOI:** 10.3390/molecules29235771

**Published:** 2024-12-06

**Authors:** Nelson Pereira, Ana Cristina Ramos, Marco Alves, Vítor D. Alves, Cristina Roseiro, Manuela Vida, Margarida Moldão, Marta Abreu

**Affiliations:** 1 Unidade de Tecnologia e Inovação, INIAV—Instituto Nacional de Investigação Agrária e Veterinária, 2780-157 Oeiras, Portugal; isa128286@isa.ulisboa.pt (N.P.); cristina.ramos@iniav.pt (A.C.R.); cristina.roseiro@iniav.pt (C.R.); manuela.vida@iniav.pt (M.V.); 2 LEAF—Linking Landscape, Environment, Agriculture and Food Research Center, Instituto Superior de Agronomia, Universidade de Lisboa, 1349-017 Lisboa, Portugal; vitoralves@isa.utl.pt (V.D.A.); mmoldao@isa.utl.pt (M.M.); 3 GeoBioTec—Geobiociências, Geoengenharias e Geotecnologias, NOVA School of Science and Technology, Universidade Nova de Lisboa, 2829-516 Caparica, Portugal; 4 INOV.LINEA/TAGUSVALLEY—Science and Technology Park, 2200-062 Abrantes, Portugal; marco_alves@tagusvalley.pt; 5 Associate Laboratory TERRA, Instituto Superior de Agronomia, Universidade de Lisboa, 1349-017 Lisboa, Portugal

**Keywords:** hot-air drying, freeze-drying, phytochemicals, carotenoids, anthocyanins

## Abstract

Sweet potato (*Ipomoea batatas* (L.) Lam.) is a nutrient-dense crop rich in fibre, minerals, and antioxidant compounds, including carotenoids and phenolic compounds, such as anthocyanins. Dehydrating sweet potato (SP) for flour production enhances its value and produces shelf-stable, health-promoting food products. This study investigated the effects of hot-air drying (HAD: 75 °C/20 h) and freeze-drying (FD: −41–30 °C/70 h) on the bioactive composition of flours from three SP varieties: *Bonita* (white-fleshed), *Bellevue* (orange-fleshed), and *NP1648* (purple-fleshed). Key assessments included the total phenolic content (TPC), the total carotenoid content (TCC), and the total anthocyanin content (TAC) and the antioxidant activity (DPPH and FRAP). The results revealed distinct raw materials’ bioactive profiles: *Bellevue* was rich in TCC (49.3 mg of β-carotene/100 g db), *NP1648* showed elevated TAC (27.3 mg of cyanidin-3-glucoside/100 g db), and *Bonita* exhibited minimal content of bioactive compounds. Both drying methods yielded significant losses of bioactive compounds, with the TPC decreasing by over 60%, while TAC and TCC losses did not exceed 32%, revealing higher stability. Multivariate analysis indicated that the variety significantly influenced the bioactive profiles more than the drying method. The interaction between carotenoids and anthocyanins and the SP fibrous composition likely contributed to their stability during drying, indicating that FD showed no advantages over HAD. The appealing colours and high antioxidant content of *Bellevue* and *NP1648* flours suggest their potential as ingredients for enhancing foods’ bioactivity and sensory acceptance.

## 1. Introduction

Sweet potato (*Ipomoea batatas* (L.) Lam.) is a tuberous root belonging to the Convolvulaceae family. It has an herbaceous consistency and exhibits favourable agronomic characteristics, including good resistance to drought. This culture exhibits a wide genetic diversity, with varieties that differ in morphological characteristics, such as the pulp and peel’s colour, the root’s shape and size, and the bioactive composition [1]. The sweet potato (SP) market in the EU has been mainly driven by the commercialisation of the whole root for fresh consumption, accounting for 90% to 92% of transactions. In recent years, Portugal has emerged as the second-largest producer of SP in Europe, just behind Spain [2]. The post-harvest conservation of SP represents a critical phase that can result in significant economic losses. This issue is closely associated with the high moisture content of the root (75% to 85%) and the delicacy of the skin, which is highly vulnerable to damage during harvesting. Therefore, the curing phase, characterised by controlled temperature and humidity conditions, plays a crucial role in post-harvest treatment, preventing the rapid deterioration of the tuber. Additionally, it is crucial to ensure adequate transport and packaging conditions to minimise the post-harvest loss of fresh commercial tubers.

The composition of SP has gained significant prominence as a relevant raw material within the food industry due to the presence of non-nutrient phytochemicals. These include phenolic compounds (e.g., flavonoids and anthocyanins), carotenoids (e.g., β-carotene), vitamins (B1, B6, C, and E), dietary fibre, and minerals (calcium, magnesium, phosphorus, and potassium) [3]. SP offers several benefits for human health, including antioxidant, anti-inflammatory, and prebiotic functions. Consumption of SP has been associated with a reduction in the risk of chronic diseases, such as cardiovascular disease, diabetes, and cancer, as well as the promotion of digestive and immune health.

Besides its widespread fresh consumption, SP can also be employed as an ingredient in diverse products, showcasing its notable versatility within the food industry. This vegetable matrix has been researched for its nutritional and bioactive richness and functional properties, and it can also act as a colouring, thickening, or gelling agent [4,5]. Therefore, SP processing into flour has gained relevance within the food industry, as it provides a novel opportunity to overcome post-harvest constraints and surplus generation. This valorisation strategy could potentially reduce food waste, increase agricultural production, and develop new highly nutritious and health-promoting ingredients or food products with the benefit of extended shelf life [6]. As a substitute for cereal-based flour, SP flour also offers the advantage of being gluten-free.

The sensory attributes (colour, flavour) and bioactive profiles of SP flours ultimately depend on genetic diversity and drying technologies, which are crucial in modulating their final characteristics and potential food applications [7]. The most reported drying method in producing vegetable flour is hot-air drying (HAD) based on convective heating, given its simplicity and cost-effectiveness [8,9]. It produces dehydrated products with an extended shelf life despite possible quality degradation due to lengthy drying times [8]. On the other hand, freeze-drying (FD) is a process based on the sublimation of frozen products, and it is frequently used to preserve the bioactive composition of high-value products, namely heat-sensitive compounds [8]. The stability of phytochemicals depends on several factors, including the operational parameters of each method (such as the temperature, power, pressure, relative humidity, and airflow rate), any pre-treatment requirements (e.g., hot water blanching), and intrinsic factors of the matrix (such as the plant species, anatomical structure, size, and shape) [8,10]. The complex interaction of phytochemicals with macronutrients in the food matrix, such as proteins, carbohydrates, and lipids, also represents a crucial factor. These nutrients can act as protective agents for phytochemicals, thereby preserving their activity during processing. An example is the interaction between antioxidant compounds and dietary fibres, which can increase antioxidant stability and effectiveness in the final food product.

This study aimed to assess the influence of various sweet potato varieties, differing in phenotype and composition, and two drying methods (hot air and freeze-drying) on the bioactive composition of gluten-free flours to optimise their bioactive potential.

## 2. Results and Discussion

### 2.1. Characterisation of the Raw Material

The initial characterisation of the raw materials is shown in Table 1.

All SP varieties revealed a high starch content (>45%), decreasing (*p* < 0.05) in the following order: *Bonita* > *Bellevue* > *NP1648*. Moisture content was notably high (>76%), with *Bellevue* exhibiting the highest value (*ca*. 83%). These contents are consistent with previously reported data for similarly coloured varieties [11]. The varieties displayed visible differences in pulp colour, instrumentally characterised using Hue angle values (Table 1): *Bonita* as white-fleshed, *Bellevue* as orange-fleshed, and *NP1648* as purple-fleshed. The bioactive composition varied significantly among varieties, with *Bellevue* showing the highest total carotenoid content and *NP1648* the highest total anthocyanin content, which correlated positively with their antioxidant activities (DPPH and FRAP methods). Similar results were reported by Teow et al. [12] and Park et al. [13] for similarly coloured varieties. The contamination levels for total viable microorganisms at 30 °C and yeasts and moulds were within the acceptable range for SP raw material [14].

### 2.2. CIELab Colour

**Figure 1:**
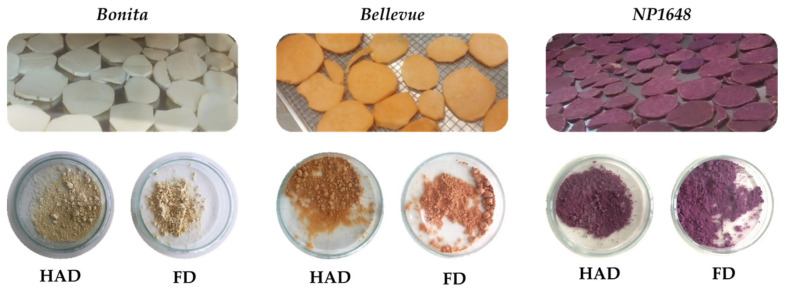
Colour of fresh samples (first row) and corresponding flours (second row) for each variety.

As illustrated in Figure 1, the processing of SPs into flour resulted in notable alterations to their original colour, irrespective of the SP variety and the drying method. These colour changes were indicated by the high values of the total colour differences (ΔE) (≥10; Table 2) and were assessed across all parameters, including hue (°h), chromaticity (Chroma), and the whitening index (WI). However, the contribution of each parameter to the colour change differed between varieties, as evidenced by the correlations with the ΔE values.

For the *Bellevue* samples, HAD maintained the orange hue (°h = 75.1; Table 2), whereas FD yielded a salmon hue (°h = 47.7; Table 2). In this variety, the freeze-dried samples had higher (*p* < 0.05) WI values than hot-air-dried samples. Conversely, the freeze-dried samples exhibited lower saturation values, indicating reduced colour purity. In the *Bonita* samples, WI (r = 0.97) and Chroma (r = −0.93) were the parameters that most contributed (*p* < 0.05) to colour change, following a similar trend to the *Bellevue* samples. For the *NP1648* samples, changes in the tone of purple (hue angle; r = 0.99) and variations in WI (r = 0.89) were the main factors responsible for colour modification (*p* < 0.05), with freeze-dried samples showing lower WI values. According to Ahmed et al. [15], colour changes in SP flour due to the drying process can usually be attributed to either the degradation of natural pigments (carotenoids and anthocyanins) or enzymatic oxidation catalysed by polyphenol oxidase (PPO). The enzymatic degradation occurs primarily after slicing, where the induction of PPO through cutting and oxygen exposure promotes darkening reactions. This process involves the degradation of phenolic compounds and the subsequent formation of dark pigments. In addition, during drying, especially at higher temperatures and longer times, Maillard reactions between the amino acids and reducing sugars in the SP can lead to browning [16]. As highlighted by Ahmed et al. [15] and Desale et al. [17], the more significant browning (indicated by lower WI values) observed in freeze-dried SP flours, compared to hot air-dried flours, can be attributed to the more effective inactivation of PPO during the HAD, in which higher temperatures were involved. In the current study, this justification only helps explain the WI changes observed for the *NP1648* variety.

### 2.3. Proximate Composition and Mineral Content

The proximate composition of all flours is shown in Table 3.

The final moisture content of all flours ranged from 2.0 to 5.3% (Table 3), and it was variety-dependent, with *Bellevue* having the highest values and *NP1648* the lowest. Similar findings were reported by Ahmed et al. [15] and Desale et al. [17] for different coloured SP flours (yellow, orange, and purple-fleshed). The final moisture content of all flours was markedly below the recommended threshold for long-term storage (<7%, [18]), which is crucial for the preservation of such products [19]. Moreover, according to the FAO Standard for Wheat Flour [18], wheat flour’s maximum water content requirement is 15%, which shows that the water content of this vegetal flour meets the standard requirements [20]. Several studies have reported similar moisture content values for SP flours derived from varieties with identical colouration. The range of reported values was 2.2–5.6% for white-fleshed, 3.4–4.4% for orange-fleshed, and 2.5–4.7% for purple-fleshed varieties [15,17,20,21,22,23,24,25]. The flours’ proximate composition remained unvaried regardless of the drying method employed; however, discernible differences were identified between varieties (Table 3). Castro-Mendonza et al. [22] reported a comparable outcome in a similar study wherein hot-air-dried and freeze-dried SP flours from two distinct varieties were compared. Similarly, this behaviour has been observed for other drying studies in other matrices, as evidenced by Silva et al. [26] and Zubia et al. [27], who studied grape skins and bignay by-products, respectively. High carbohydrate content (>85%), as well as low fat (<1%) and protein (≤6%) values, were found to be characteristic of SP root. *Bellevue* stood out with the lowest carbohydrate content (*p* < 0.05) and the highest fat content (*p* < 0.05) compared to the others. The protein content differed (*p* < 0.05) between varieties in the following order: *NP1648* > *Bellevue* > *Bonita*. These values fall within the range of variation reported in other studies investigating SP flours from varieties with identical colouration: 1.5–4.2% for white-fleshed, 2.1–4.3% for orange-fleshed, and 1.6–8.4% for purple-fleshed varieties. The fat content of SP flours did not exceed the values reported in other similar studies (<2%), regardless of the variety [15,17,20,21,22,23,24,25]. Despite significant differences between varieties, the ash content did not surpass 4%, decreasing in the following order: *Bellevue* > *Bonita* > *NP1648*. Ash content values within a similar range (1.9% to 4.3%) have been reported in studies investigating SP flours derived from different-coloured varieties [15,17,22,28].

Table 4 shows the most relevant minerals quantified in SP flours (levels > 1%). The remaining minerals (Si, Fe, Cu, Zn, Mn, B, Mo, Cr, Ni, Cd, and Pb) were present in trace amounts (levels < 0.02%) or not identified.

The flour’s mineral composition was not affected by the drying method employed. The observed differences between varieties can be attributed to their intrinsic characteristics. Potassium was the most abundant mineral in all of the flours, with the *Bellevue* showing the highest levels (*ca*. 26%), followed by *Bonita* (*ca*. 22%) and *NP1648* (*ca*. 16%). On the other hand, *Bellevue* exhibited the lowest levels of sodium (*ca*. 0.7%) and magnesium (*ca*. 1.0%), while *NP-1468* had the highest levels of calcium (*ca.* 2.8%) and phosphorus (*ca.* 3.4%). The sulphur content (*ca*. 1.5%) was similar for all varieties. No heavy metals (Cd and Pb) were detected in any of the SP flours analysed, irrespective of the drying method or variety. The absence of heavy metals in flours intended for human consumption represents a significant advantage regarding food safety. Xu et al. [23] reported that FD did not alter the macronutrient content of SP flour. Mihály-Lango et al. [25] further noted that variations in mineral content among different flours were predominantly attributed to genetic differences, with the purple-fleshed variety showing the highest levels of minerals (Mg, K, Na, Se, Mn, and Cu). In freeze-dried flours derived from orange and yellow-fleshed SP varieties, Zhang et al. [11] also reported high concentrations of K and P, moderate amounts of Mg and Ca, and trace levels of Fe, Zn, Cu, Mn, Cr, Ni, Se, and Cd. The mineral content reported in this study was higher than those observed in the present study, a discrepancy that could be attributed to SP genetic differences and drying conditions.

### 2.4. Bioactive Composition

Table 5 shows the bioactive composition of fresh SP and hot-air-dried and freeze-dried flours from the three studied varieties.

The total phenolic content (TPC) in the flours exhibited a notable influence from the drying method (f ≈ 390) and the SP variety (f ≈ 139). TPC losses were substantial, exceeding 60% in *Bellevue* flours and 71% in *NP1648* flours, regardless of the drying process. However, the TPC losses in *Bonita* flour exhibited distinct behaviour between the drying methods, as a 69% reduction was observed with HAD, while the initial values were retained with FD. Concerning freeze-dried SP flours from similarly coloured varieties, Huang et al. [29] reported TPC values ranging from 10.1 to 80.8 mg of GAE/100 g db, which is comparable to the present study’s results (Table 5). However, other studies have reported lower TPC values (e.g., 0.1–42.2 mg of GAE/100 g db [12] and 14–43 mg of GAE/100 g db [30]), while others have reported higher TPC values (301.5–362.8 mg of GAE/100 g db [31]). Despite these variations, the results show that the phenolic composition in SP is considerably sensitive to the drying process, even when carried out through FD. In a study by Belkacemi et al. [32] involving white-fleshed SP hot-air-dried flour (60 °C/24 h), the reported TPC values (53.15–132.58 mg of GAE/100 g) were higher than those observed in the hot-air-dried flours in the present study (Table 4). Additionally, Belkacemi et al. [32] compared flours processed with and without thermal blanching (100 °C/5 min) before drying, revealing a significant TPC reduction in the blanched samples. This finding underscores the heat sensitivity of these phytochemicals. A direct comparison of the TPC losses induced by different drying methods in SP flours was not feasible, as most studies did not provide the raw material TPC and necessary quantification. Moreover, the variability in TPC across similar studies can be attributed to genetic differences among SP varieties and the influence of diverse agricultural practices and soil- and climate-related factors, as documented by Oke and Workneh [33]. On the other hand, drying studies conducted on various plant matrices have generally shown that TPC losses induced by drying—regardless of the method used—are comparable to the values quantified in the present study. Silva et al. [26] observed a 69% reduction in TPC in grape skin residues compared to fresh samples, regardless of the drying method (HAD vs. FD). Similarly, Akcikek et al. [34] and Kayacan et al. [35] reported TPC reductions of over 70% in blueberry and persimmon samples dried using hot air, respectively. Studies on pomegranate arils [36], bignay by-products [27], star fruit [16], and pomelo by-products [37] have also noted TPC losses after HAD and FD, although to a lesser extent (not exceeding 50%). These findings highlight again the significant heat sensitivity of phenolic compounds during drying.

Regarding the total carotenoid content (TCC) of the flours, the effect of the SP variety was substantially more pronounced (f ≈ 6063) than the effect of the drying method (f ≈ 85). A similar pattern was observed for the flours’ β-carotene content. The *Bellevue* flours exhibited the highest TCC and β-carotene content (Table 5), regardless of the drying method. In contrast, the other varieties displayed marginal TCC and β-carotene levels. The carotenoid profiles indicated that β-carotene was the predominant carotenoid present in the orange-fleshed variety, also shown by a strong positive correlation with the TCC (r = 0.97; *p* < 0.05). For the *Bellevue* variety, the drying method significantly impacted the TCC, with HAD leading to a 32% decrease compared to a 12% reduction with FD. Similarly, the β-carotene content showed a more pronounced decline with HAD (*ca.* 53%) than with FD (*ca.* 28%). Vimala et al. [38] reported that orange-fleshed SP flours retained a high proportion of carotenoids during oven drying, with 89–96% retention rates, indicating minimal degradation during drying. These retention levels are crucial, as β-carotene imparts the characteristic deep orange colour to SP and significantly enhances their nutritional and bioactive value. However, significant TCC losses have been reported in other plant matrices. Fernandes et al. [39] observed a TCC decrease exceeding 70% for edible *Centaurea* petals dried through HAD at 50 °C and FD. Conversely, Kayacan et al. [35] found that FD effectively retained the β-carotene content of persimmon, while HAD at 55 °C yielded a 33% decrease. Variations in TCC retention may be attributed to differences in enzymatic oxidation during processing [38]. Moreover, compared to other plant matrices, the lower losses of TCC observed in SP flours can be attributed to the interaction of carotenoids with other components of the SP, namely starch and fibres, which may act as protective factors [9,40,41].

The total anthocyanin content (TAC) of the flours was significantly influenced by the SP variety (f ≈ 527) rather than by the drying method (f ≈ 69). The *NP1648* flours exhibited the highest TAC values (Table 5), regardless of the drying method, which was expected due to its purple pigmentation. Similar findings have been reported by Amagloh et al. [4] and Teow et al. [12]. However, the drying method significantly affected flour’s TAC, with higher losses observed through FD (*ca.* 32%) compared to HAD (*ca.* 20%). The drying method also impacted the TAC of *Bellevue* flours, with losses ca. three times higher using HAD (*ca.* 66%) than FD (*ca.* 23%). In contrast, the *Bonita* flours retained the low TAC levels found in the raw material (Table 5) irrespective of the drying method. Overall, the TAC exhibited intermediate stability during drying compared to the TPC and the TCC. These TAC losses in SP flours are consistent with findings of other studies. For instance, Ozay-Arancioglu et al. [36] reported a 55% loss in hot-air-dried pomegranate arils, while Fernandes et al. [39] found losses between 34 and 44% in edible *Centaurea* petals dried through HAD and FD.

The flours’ antioxidant activity (AOx), as determined using the FRAP method, exhibited variations similar to those observed for the TPC, with both the drying method (f ≈ 1774) and the SP variety (f ≈ 1335) exerting a significant influence. For the *Bonita* variety, a substantial AOx loss (59%) was observed in hot-air-dried flours, whereas freeze-dried flours retained AOx contents similar to those of the raw material (Table 5). In contrast, for the other varieties, HAD yielded significantly higher losses (*ca*. 67%) compared to FD (*ca*. 58%). Moreover, a strong positive correlation (r = 0.98; *p* < 0.05) was found between FRAP and TPC, underscoring that phenolic compounds may be the main contributor to AOx across all varieties. A comparable study conducted by Castro-Mendonza et al. [22] on two SP varieties dried through HAD and FD reported that purple SP flour consistently exhibited higher FRAP values than yellow SP flour, regardless of the drying method. Furthermore, the study found that for the purple-fleshed variety, the freeze-dried flours displayed higher FRAP values (78.0 mmol TE/g db) compared to the hot-air-dried flours (22.7 mmol TE/g db), as corroborated by the present study. Amagloh et al. [4] also reported a wide range of FRAP values across freeze-dried SP flours of varying colours, with the orange-fleshed varieties exhibiting the highest FRAP values. Other studies suggest that FRAP losses may vary according to the plant matrix and the drying method. Dhara et al. [16] found FRAP losses exceeding 80% in dried star fruit, regardless of the drying method (HAD vs. FD). For grape skin residues [26], FD yielded a 55% loss, while HAD yielded a 11% loss. Small FRAP losses were also reported for hot-air-dried black grapes (14%; [42]) and bignay by-products (13%; [27]).

The evaluation of AOx using the DPPH method, which distinguishes between hydrophilic and lipid fractions, revealed a significant influence of both the drying method and the SP variety. These effects were more pronounced in the hydrophilic fractions, with f ≈ 2794 for the drying method and f ≈ 620 for the SP variety, than in the lipid fractions, where f ≈ 60 and f ≈ 41 were observed, respectively. The high f values indicate that the AOx of the hydrophilic fraction exhibited greater variations induced by the drying method than by the SP variety. Regardless of the drying method employed, a reduction in AOx using the DPPH method was observed (Table 5). The AOx changes were significantly more pronounced in the hydrophilic fraction, with losses exceeding 70%, compared to the lipid fraction, which denoted losses below 60%. For the hydrophilic fraction, no differences were observed between drying methods for *Bellevue* and *NP1648* varieties, with average losses exceeding 75%. In contrast, for the *Bonita* variety, HAD yielded a higher degradation of hydrophilic antioxidant compounds (*ca*. 72%) than FD (*ca*. 33%). This trend was similar to that observed for the TPC, which was supported by a high positive correlation (r = 0.92, *p* < 0.05). Regarding the lipid fraction, no significant differences were observed between the drying methods across all flours. The lowest losses were observed for *NP1648* (*ca*. 38%), followed by *Bellevue* (*ca*. 44%) and *Bonita* (*ca*. 58%). Teow et al. [12] and Rumbaoa et al. [43] compared the bioactive composition of freeze-dried SP flours derived from purple-, white-, and yellow-fleshed varieties, reporting that the purple flours exhibited higher DPPH radical scavenging activity compared to white- and yellow-fleshed varieties, as observed in the present study. Moreover, Teow et al. [12] identified phenolic compounds and anthocyanins as the primary contributors to the hydrophilic antioxidant activity, whereas carotenoids and tocopherols were identified as the primary antioxidants in lipid fractions. High percentages of AOx loss (DPPH method—hydrophilic fraction) have also been reported in various plant matrices. A 56% decrease in AOx was found using the DPPH method for hot-air-dried blueberries [34], a 66% decrease for hot-air-dried persimmon [35], and 55–65% decreases in grape skin dried through HAD and FD [26]. Other studies reported smaller losses (*ca*. 26%) in hot-air-dried pomegranate arils [36] and hot-air-dried black grapes [42].

### 2.5. Microbial Counts

**Figure 2:**
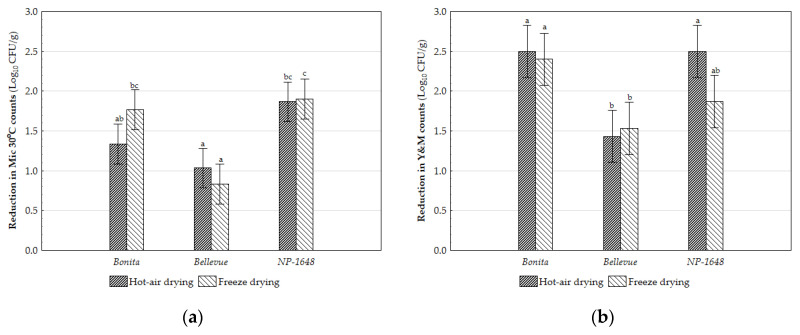
Reduction levels of total microorganism ((**a**) log_10_ CFU/g) and Y&M ((**b**) log_10_ CFU/g) counts for hot-air-dried and freeze-dried SP flours from the three studied varieties compared to the raw material. Vertical bars denote 0.95 confidence intervals. Means sharing letters are not significantly different; different letters indicate *p* < 0.05 (Tukey).

The drying process reduced the microbial levels for both microbiological groups, with no significant differences between HAD and FD, regardless of the variety (Figure 2). However, the mesophilic microorganisms showed higher resistance to the drying process (*ca*. 1.5 log_10_ CFU/g) than the yeast and mould (Y&M) group (*ca.* 2.0 log_10_ CFU/g). The average values of mesophilic microorganisms in the different flours obtained through HAD (between 3.4 and 3.4 log_10_ CFU/g) and FD (between 3.0 and 4.2 log_10_ CFU/g) were below the limits allowed for this type of product (≤5 log_10_ CFU/g; [14]). A similar result is reported for the Y&M group, where levels were lower than 4 log_10_ CFU/g [14], as shown by the values obtained in the hot-air-dried flours (between 1.2 and 2.1 log_10_ CFU/g) and in the freeze-dried flours (*ca*. 2.0 log_10_ CFU/g). The reduced microbial counts, combined with the low water activity (a_w_) levels of the flours (<0.4), suggest the long-term preservation of these food ingredients, as it has been well established that an a_w_ below 0.6 can inhibit the susceptibility of food products to microbial spoilage [44].

### 2.6. PCA Modelling

To better visualise the interaction between the effects of the drying method and the SP variety on flour composition, a multivariate analysis (PCA and hierarchical clustering) was carried out. A preliminary analysis was conducted to identify which quantitative variables significantly contributed to the model. Variables, such as microbial counts and CIELab colour parameters, were excluded due to low factor loadings, while a_w_ was excluded due to autocorrelation with moisture content. The final data matrix comprised 12 quantitative variables and 18 samples, categorised by SP variety (*Bonita*—‘Bon’; *Bellevue*—‘Bell’; and *NP1648*—‘NP’) and drying method (HAD and FD). The selected variables (loading factors are provided in Supplementary Material Table S1) included TPC, DPPH (MeOH and DCM), FRAP, TCC, β-carotene content, TAC, and proximate composition (moisture, ash, fat, protein, and carbohydrate contents).

**Figure 3:**
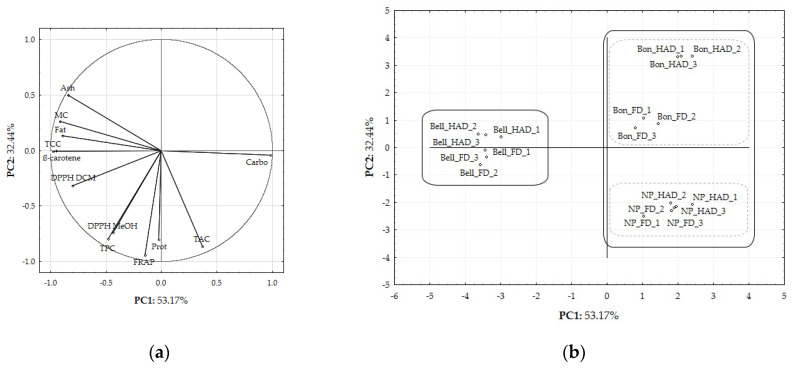
Principal component analysis (PCA) of the composition of SP flours, influenced by variety and drying method: (**a**) loading plot and (**b**) score plot. Abbreviations: TPC-total phenolic content; TCC-total carotenoid content; DPPH MeOH-AOx through the DPPH method for the hydrophilic fraction; DPPH DCM-AOx through the DPPH method for the lipid fraction; FRAP-AOx through the FRAP method; TAC-total anthocyanin content; β-carotene-β-carotene content; MC-moisture content; Ash-ash content; Prot-protein content; Fat-fat content; Carbo-carbohydrate content.

Figure 3 shows the spatial projection plots, illustrating the ordination of the variable vectors (a) and the distribution of the samples (b) in the plane defined by the first two principal components. The PCA identified two principal components—PC1 and PC2—that accounted for 85.61% of the total variance in the dataset.

The dominant variables for the first principal component (PC1), which explained 53.17% of the total variance, included moisture, fat, ash, and carbohydrate content, along with the lipophilic antioxidant composition (DPPH DCM, TCC, and β-carotene content). All variables were negatively correlated with PC1, except for the carbohydrate content. The second principal component (PC2), accounting for 32.44% of the total variance, was mainly influenced by the hydrophilic antioxidant composition (TPC, DPPH MeOH, FRAP, and TAC) and the protein content, with all variables negatively correlated with PC2. The score plot (Figure 3b) revealed sample groupings consistent with the hierarchical cluster dendrogram (Supplementary Material Figure S1), highlighting a clear differentiation in SP flours’ proximal and bioactive compositions. The positioning of samples along PC1, from right to left, corresponded to an increase in lipophilic antioxidant compounds, fat, and ash content and a decrease in carbohydrate content. In contrast, the positioning along PC2, from top to bottom, reflected increasing levels of hydrophilic antioxidant compounds (phenolics and anthocyanins) and protein content. The first division in the hierarchical clustering (linkage distance < 7) separated *Bellevue* flours from the other varieties, mainly due to their TCC and carbohydrate content, independent of the drying method applied. The second division (linkage distance < 5) further distinguished the *Bonita* and *NP1648* varieties based on the presence of hydrophilic antioxidant compounds, regardless of the drying method. The separation between FD and HAD samples is more pronounced in the *Bonita* variety than in the others. This fact can be attributed to the significant retention of TPC in FD compared to HAD, which was not observed in the remaining varieties.

*Bellevue* flours stood out for their high TCC, particularly their β-carotene content, contributing to their high lipophilic antioxidant activity (DPPH DCM) and fat and ash content. In contrast, *NP1648* flours were notable for their high TAC, reflected in their high hydrophilic antioxidant activity (DPPH MeOH) and carbohydrate content. *Bonita* flours (FD and HAD) also exhibited a high carbohydrate content but reduced levels of bioactive compounds. These results underscored the central role of varietal selection in shaping the nutritional and antioxidant profiles of the SP flours, highlighting *Bellevue* and *NP1648* varieties for their bioactive potential. The drying method also played a significant role (Table 5). While FD is generally associated with better retention of bioactive compounds, this was only observed in the current study for TCC in *Bellevue* flours. Conversely, HAD contributed to higher TAC retention in *NP1648* flour than FD. Despite the differences mentioned above, the carotenoid content in both hot-air-dried and freeze-dried flours (ranging from 33.5 to 43.6 mg of β-carotene/100 g db, respectively; Table 5) remained within the nutritionally relevant range concerning the recommended daily intake of β-carotene (700–900 mcg RAE, which is equivalent to 8.4–10.8 mg of β-carotene) [45]. Therefore, the higher costs associated with FD may not be justified.

## 3. Materials and Methods

### 3.1. Plant Material and Sweet Potato Flour Preparation

**Figure 4:**
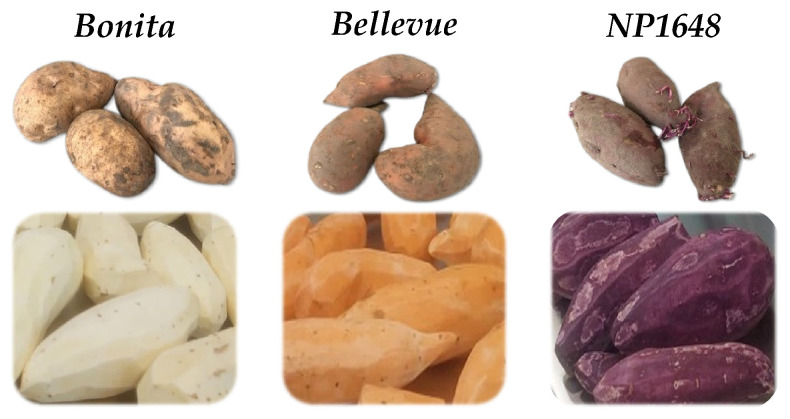
Varieties under study: *Bonita* (white-fleshed), *Bellevue* (orange-fleshed), and *NP1648* (purple-fleshed).

The sweet potato varieties employed in this study were cultivated in Portugal by the company NativaLand and selected for their phenotypic and compositional diversity and capacity to adapt well to the national edaphoclimatic conditions. The varieties under study were *Bonita* (white-fleshed), *Bellevue* (orange-fleshed), and *NP1648* (purple-fleshed) (Figure 4). The varieties were harvested in 2023 and underwent a curing process (temperature of 30 °C; 95% relative humidity; suitable ventilation; 2–7 days) as a technique to heal minor cuts and wounds on the root surface and improve post-harvest storage. Subsequently, the SP varieties were stored under controlled temperatures (12–15 °C), relative humidity (80%), and ventilation conditions for up to one week. Subsequently, the raw material was transported to the INIAV laboratory.

The experimental design used for this study was a 2 × 3 full factorial design considering two factors, drying method (HAD and FD) and SP variety (*Bonita*, *Bellevue*, and *NP1648*), resulting in 6 experimental combinations and a total of 18 samples. This design allowed for assessing the factors’ main effects and potential interactions.

The transformation into flour (drying and milling) was conducted at the Tagus Valley Science and Technology Park (Abrantes, Portugal). All SP varieties were cut into 4 mm slices and subjected to two drying methods: hot-air drying at 75 °C for 20 h (Klarstein Master Jerky 32, Berlin, Germany), according to previous studies in sweet potato [9], and freeze-drying at a temperature range of −41 to 30 °C for 70 h (Coolvacuum Lyobiotic 10FD, Barcelona, Spain). The dried samples were milled into flour using a food processor (Vorwerk, Wuppertal, Germany; maximum speed for 1 min) and then sieved through a 250 µm mesh. All flours were vacuum-packed using LPDE films and stored at −80 °C until further analysis. The flour samples (n = 18) and the raw material were characterised following the procedures outlined in Section 3.2 and Section 3.3.

### 3.2. Analytical Procedures

#### 3.2.1. Starch Content

With slight modifications, the starch content was determined according to Zavareze et al. [46]. The samples were ground in water in a 1:1 (*m*/*v*) ratio, filtered through cloth, and incubated at room temperature for 24 h to allow for decantation and precipitate formation. Afterwards, the supernatant was carefully removed, and the precipitate was washed with water to ensure starch quality. The samples were then dried at 35 °C for 12 h (Heraeus D-6450, Hanau, Germany).

#### 3.2.2. Proximate Composition and Mineral Content

The flours were analysed in triplicate for their moisture, ash, protein, and fat content. Moisture content was determined according to NP-875 (1994) [47] at 105 °C (Heraeus D-6450, Hanau, Germany), and a_w_ was determined with Rotronic, Hygrolab equipment. The ash content was determined using the muffle furnace (Thermolyne 48000 Muffle Furnace, Thermo Fisher Scientific, Waltham, MA, USA) according to the official method 923.03 [48]. The protein content was determined using a Dumas Nitrogen Analyzer (VELP Scientific NDA 702 DUMAS Nitrogen Analyzer—TCD detector, Usmate Velate, Italy). The protein content was obtained by multiplying the total nitrogen content by the conversion factor 6.25. The fat content was determined according to Akbar et al. [49], with slight modifications. A mass of 2 g of each flour was extracted with 150 mL of n-hexane (Honeywell, Charlotte, NC, USA) in a Soxhlet apparatus for 3 h. The solvent was removed using a rotary evaporator (Heidolph G1, Schwabach, Germany) equipped with a vacuum pump (MZ 2C NT, Vacuubrand GMBH, Wertheim, Germany), and the residue was dried in an oven at 60 °C overnight. The carbohydrate content was estimated by difference, as follows: % Carbohydrate = 100 − (% moisture + % protein + % fat + % ash).

The mineral content was determined according to Leitão et al. [50]. About 0.5 g of each flour (triplicates) was digested in a solution composed of 2 mL of a 65% nitric acid solution (Sigma-Aldrich, St. Louis, MO, USA) and 6 mL of a 37% hydrochloric acid solution (Chem-Lab, Zedelgem, Belgium). Digestion occurred in a heating block (DigiPrep, MS 50 mL of 48 Pos digester, SCP Science, Baie-D’Urfe, QC, Canada) for 1 h at 105 °C. After digestion, the volumes of the solutions were adjusted to 50 mL with distilled water. The solutions were homogenised and allowed to settle. The determination of mineral elements (Na, K, Ca, Mg, P, S, Si, Fe, Cu, Zn, Mn, B, Mo, Cr, Ni, Cd, and Pb) was performed using ICP-OES (iCAP 7000 series, Thermo Scientific, Waltham, MA, USA).

#### 3.2.3. CIELab Colour Measurements

A colourimeter (Minolta CR-300, Osaka, Japan) was used to evaluate the samples’ colour by measuring L* (0: black; 100: white), a* (−60: green; +60: red), and b* (−60: blue; +60: yellow) parameters (C illuminant, second observer). The instrument was calibrated using a white tile standard (L* = 97.10; a* = 0.19; b* = 1.95). The chroma index (C*), hue angle (°h), whiteness index (WI), and total colour difference (∆E) were calculated as follows:

(1) $$C^{*}=\sqrt{\left(a^{*2}+b^{*2}\right)}$$

(2) $${}^{\circ}h=\tan^{-1}\left(\frac{b^{*}}{a^{*}}\right)$$

(3) $$WI=100-\sqrt{{\left(100-L^{*}\right)}^{2}+{a^{*}}^{2}+{b^{*}}^{2}}$$

(4) $$∆E=\sqrt{{\left(L^{*}-L_{0}^{*}\right)}^{2}+{\left(a^{*}-a_{0}^{*}\right)}^{2}+{\left(b^{*}-b_{0}^{*}\right)}^{2}}$$

#### 3.2.4. Extract Preparation for TPC and AOx Determinations

The hydrophilic fractions, used to determine the total phenolic content and antioxidant activity, were prepared from a 1:2.25 (*m*:*v*) mixture of the sample with methanol (100%; Honeywell, Charlotte, NC, USA) homogenised at 20,000 rpm for 1 min (Polytron Ultra-Turrax T 25 basic, IKA-Werke, Staufen, Germany) and incubated for 20 min in an ultrasonic bath (Sotel Branson 2200 Ultrasonic Cleaner, Bayern, Germany). Centrifugation followed at 7000 rpm for 20 min at 4 °C (Sorvall RC5C, SS34 rotor, Sorvall Instruments, Wilmington, DE, USA), and the supernatant was collected and stored at −20 °C (Large Upright AEG OKO_ARCTIS Freezer, Los Angeles, CA, USA) until analysis. The lipid fractions, used to determine the total carotenoid (TCC) and the β-carotene content, were prepared as described, with dichloromethane instead of methanol.

#### 3.2.5. Total Phenolic Content

The total phenolic content was determined as described by Swain et al. [51], with slight modifications. In total, 2400 μL of distilled water, 150 μL of extract, and 150 μL of 0.25 M Folin-Ciocalteu reagent (Sigma-Aldrich, St. Louis, MO, USA) were mixed and stirred in test tubes. Then, 300 μL of 1 M sodium carbonate (Merck Millipore, Burlington, MA, USA) was added, followed by 2 h in the dark at room temperature. Spectrophotometric readings were taken at 725 nm (Jas.co V-530 UV/Vis Spectrophotometer, Tokyo, Japan). The TPC results were expressed in milligrams of gallic acid equivalents per 100 g of dry weight (mg GAE/100 g db).

#### 3.2.6. Antioxidant Activity (DPPH and FRAP Methods)

The antioxidant activity determined using the DPPH method was based on the procedures described by Arnao et al. [52] and Brand-Williams et al. [53], with some modifications. First, 150 µL of extract and 2850 µL of DPPH solution (TCI Chemicals, Zwijndrecht, Belgium) were mixed and incubated at room temperature for 2 h in the dark. The absorbance was measured spectrophotometrically at 515 nm (Jas.co V-530 UV/Vis Spectrophotometer, Japan). The results were expressed in µmol of Trolox equivalents per 100 g dry weight (µmol ET/100 g db). The AOx determined using the FRAP method was based on the procedures described by Thaipong et al. [54], with some modifications. The FRAP reagent stock solution was prepared from a mixture of 0.3 M sodium acetate buffer (pH 3.6) (Sigma-Aldrich, St. Louis, MO, USA), 10 mM TPTZ solution (Alfa Aesar, Haverhill, MA, USA), and 20 mM ferric chloride solution (Sigma-Aldrich, St. Louis, MO, USA), in a 10:1:1 ratio. Then, 0.2 mL of extract and 1.8 mL of FRAP reagent stock solution were mixed and incubated at room temperature for 5 min and then measured spectrophotometrically at 593 nm (Jas.co V-530 UV/Vis Spectrophotometer, Japan). The results were expressed as mmol of FeSO_4_·7H_2_O equivalents per 100 g of dry weight (mmol FeSO_4_·7H_2_O/100 g db).

#### 3.2.7. Total Carotenoid Content

The total carotenoid content was determined as described by Talcott and Howard [55]. About 1 g of the sample was homogenised at 20,000 rpm for 1 min (Polytron Ultra-Turrax T 25 basic, IKA-Werke, Staufen, Germany) in 20 mL of acetone: ethanol (1:1, *v*:*v*) containing 200 mg/L of BHT (extraction solution). The homogenate was washed with the extraction solution through filtration until no additional colour change was observed. Then, 50 mL of n-hexane was added and incubated for 30 min. Then, 25 mL of nano-pure water was added to allow the phases to separate, and the organic phase was collected. Spectrophotometric readings were taken at 470 nm (Jas.co V-530 UV/VIS Spectrophotometer). The results were expressed as milligrams of β-carotene equivalents per 100 g dry weight (mg β-carotene/100 g db).

#### 3.2.8. Total Anthocyanin Content

The total anthocyanin content was determined using the differential pH method described by Lee et al. [56]. Samples were diluted in distilled water in a 1:5 (*m*/*v*) ratio and homogenised (Polytron Ultra-Turrax T 25 basic, IKA-Werke) for 3 min. Then, the mixtures were placed under agitation at room temperature for 15 min, followed by centrifugation at 3000 rpm for 10 min at 4 °C. The supernatant was collected and filtered with 150 mm diameter qualitative filter paper discs. About 1 mL of each extract was added to two test tubes containing 9 mL of buffer solution at pH 1 and pH 4.5 each. After incubation for 5 min in the dark at room temperature, the absorbance values were read spectrophotometrically at 510 nm and 700 nm (Jas.co V-530 UV/VIS Spectrophotometer). The results were expressed in milligrams of cyanidin-3-glucoside equivalents per 100 g dry weight (mg cyd-3-glu/100 g db).

#### 3.2.9. β-Carotene Content

The extracts prepared in Section 3.2.7 were analysed to quantify the β-carotene content across all samples. The β-carotene content was determined using a reverse phase HPLC method in a Waters HPLC system consisting of an Alliance 2695 separation module (Waters, Milford, MA, USA) and a Photo Diode Array detector (Waters 996, Waters, Milford, MA, USA). Chromatographic separation was performed in a YMC Carotenoid column (250 × 4.6 mm ID S-5 μm, Japan) at room temperature using a gradient elution with a mixture of methanol: H_2_O (75:25) as solvent A and ethyl acetate as solvent B. The gradient elution was 70% of solvent A at 0 min, followed by a linear gradient to 10% solvent A at 20 min and a return to the initial condition by 20 min. The total run time of the analysis was 40 min, and the flow rate was 0.80 mL min^−1^. Detection was performed for the UV-PDA detector at 450 nm. Quantification was based on the external standard technique from a standard peak area vs. concentration curve.

### 3.3. Microbial Analysis

The total microorganism at 30 °C counts were carried out following ISO 4833-1:2013 [57]. Successive decimal dilutions were made in Ringer’s solution (Biokar Diagnostics, Allone, France), which was then incubated in PCA medium (Plate Count Agar; Biokar Diagnostics, Allone, France) at 30 °C for 72 h. The yeasts and mould counts were carried out following ISO 21527-1:2008 [58], using the DRBC agar culture medium (Dichloran Rose-Bengal Chlortetracycline Agar; Biokar Diagnostics, Allone, France). About 0.1 mL of each dilution prepared was transferred into the medium and incubated at 25 ± 1 °C for 5 days under aerobic conditions.

### 3.4. Statistical Analysis

Data were subjected to analysis of variance (two-factor factorial ANOVA) using Statistica^TM^ v. 8.0 Software from StatSoft [59]. Statistically significant differences (*p* < 0.05) between samples were determined according to Tukey’s HSD test. The F-value was used to assess the individual influence of each factor (SP variety and drying method) and the interaction between them on the dependent variable. A higher F-value indicates a greater contribution of that effect to the variability in the dependent variable, suggesting a stronger influence of the factors being evaluated. Pearson correlation coefficients were also determined between the studied responses. A principal component analysis (PCA) was also performed. Before analysis, all variables were mean-centred and standardised (scaled) to unit variance (correlation matrix). The principal components were obtained by computing the study data correlation matrix’s eigenvalues and eigenvectors [60]. A score for each sample was calculated as a linear combination for each PCA component. The contribution of each variable to the PCA score was deduced from the parameter loading for the factor. A bi-dimensional representation of this multi-dimensional dataset was made for the primary components that accumulated a significant amount of original information, above 70%, which is deemed sufficient to establish a good model for qualitative purposes [61]. A hierarchical cluster analysis was performed; the clustering process included data standardisation, sample dissimilarity metric assessment, and grouping technique application. The Euclidean distance was used as a dissimilarity distance, and Ward’s method was used as the grouping technique.

## 4. Conclusions

The production of sweet potato flours with high bioactivity, using different varieties and drying methods, revealed that the intrinsic characteristics of each variety played a more decisive role in defining the bioactive profiles of the flours than the drying method. The loss of bioactive compounds during drying varied according to their nature, with carotenoids and anthocyanins showing higher stability (losses < 32%) compared to total phenolic compounds (losses > 60%), regardless of the drying method. This higher stability may be associated with the interaction of carotenoids and anthocyanins with the fibrous component of sweet potato.

Due to their high carotenoid and anthocyanin content, the *Bellevue* and *NP1648* varieties emerged as optimal options for producing bioactive SP flours. These varieties have the potential to offer both nutritional and sensory benefits, mainly through their attractive colours. Furthermore, freeze-drying did not demonstrate significant advantages over hot-air drying, suggesting that the higher costs associated with FD may not be justified. Moreover, the flours produced showed low moisture content and microbial contamination levels within quality standards, ensuring product safety and long-term preservation.

These findings underscore the potential of SP flours as ingredients for developing new gluten-free foods with high bioactive value, contributing to the diversification of healthy food options.

## Figures and Tables

**Table 1:** Physico-chemical and microbiological characterisation of raw samples. For each line, different letters indicate significant differences (*p* < 0.05).

Quality Attribute	Variety		
	*Bonita*	*Bellevue*	*NP1648*
Moisture content (%)	75.8 ^a^ ± 0.3	82.6 ^b^ ± 1.0	77.0 ^a^ ± 1.9
a_w_	0.97 ^a^ ± 0.0	0.97 ^a^ ± 0.0	0.97 ^a^ ± 0.0
Starch content (%)	53.3 ^c^ ± 1.5	48.9 ^b^ ± 1.4	45.0 ^a^ ± 0.7
Pulp’s CIELab colour			
L*	86.4 ^c^ ± 0.3	72.1 ^b^ ± 0.5	38.7 ^a^ ± 1.3
a*	−3.5 ^a^ ± 0.2	24.6 ^b^ ± 1.9	24.5 ^b^ ± 0.6
b*	25.2 ^b^ ± 0.7	43.8 ^c^ ± 3.3	2.8 ^a^ ± 0.4
Croma	25.5 ^a^ ± 2.5	50.3 ^b^ ± 5.3	24.7 ^a^ ± 4.2
Hue	97.9 ^c^ ± 1.4	69.7 ^b^ ± 2.6	6.7 ^a^ ± 2.9
Total phenolic content (mg gallic acid eq./100 g db)	76.4 ^a^ ± 8.3	173.2 ^b^ ± 23.2	226.9 ^c^ ± 5.0
Antioxidant activity			
Hydrophilic DPPH (µmol trolox eq./100 g db)	12,487.5 ^a^ ± 1863.4	43,461.0 ^c^ ± 1192.7	34,359.3 ^b^ ± 159.2
Lipidic DPPH (µmol trolox eq./100 g db)	3330.8 ^a^ ± 140.5	5172.4 ^b^ ± 172.9	3106.3 ^a^ ± 193.0
FRAP (µmol FeSO_4_·7H_2_O/100 g db)	23.8 ^a^ ± 1.0	66.9 ^b^ ± 4.3	103.3 ^c^ ± 1.6
Total carotenoid content (mg β-carotene/100 g db)	0.4 ^a^ ± 0.1	49.3 ^b^ ± 2.0	1.7 ^a^ ± 0.2
β-carotene content (mg/100 g db)	n.d	27.2 ± 4.3	n.d
Total anthocyanin content (mg cyanidin-3-glucoside/100 g db)	7.4 ^a^ ± 0.7	14.9 ^b^ ± 2.4	27.3 ^c^ ± 0.7
Total microorganisms at 30 °C (log_10_ CFU/g)	4.8 ^a^ ± 0.1	5.0 ^ab^ ± 0.1	5.3 ^b^ ± 0.3
Yeasts and moulds (log_10_ CFU/g)	4.3 ^b^ ± 0.2	3.5 ^a^ ± 0.1	3.7 ^a^ ± 0.1

n.d: non-detectable.

**Table 2:** Instrumental colour evaluation (Chroma, °h, WI, and ΔE) for fresh SP, hot-air-dried, and freeze-dried flours from the three studied varieties. For each column, different letters indicate significant differences (*p* < 0.05).

Processing	Variety	Chroma	°h ^1^	WI	ΔE
Raw material	*Bonita*	25.5 ^a^ ± 2.5	97.9 ^h^ ± 1.4	71.1 ^g^ ± 2.1	-
HAD		17.5 ^bc^ ± 0.5	94.6 ^g^ ± 0.3	80.4 ^b^ ± 0.9	9.5 ^b^ ± 0.7
FD		16.0 ^b^ ± 0.5	101.5 ^i^ ± 0.2	82.6 ^b^ ± 0.8	12.0 ^c^ ± 1.0
Raw material	*Bellevue*	50.3 ^f^ ± 5.3	60.7 ^e^ ± 2.6	42.5 ^a^ ± 5.4	-
HAD		37.6 ^e^ ± 1.7	75.1 ^f^ ± 0.6	56.7 ^e^ ± 0.8	18.2 ^a^ ± 0.6
FD		32.2 ^d^ ± 0.6	47.7 ^d^ ± 0.3	61.8 ^f^ ± 1.1	21.5 ^e^ ± 0.8
Raw material	*NP1648*	24.7 ^a^ ± 4.2	6.7 ^b^ ± 2.9	33.8 ^c^ ± 4.2	-
HAD		19.0 ^c^ ± 0.7	5.0 ^a^ ± 0.3	50.1 ^d^ ± 1.1	16.8 ^d^ ± 1.0
FD		26.3 ^a^ ± 0.6	20.3 ^c^ ± 0.3	45.1 ^a^ ± 1.5	17.8 ^a^ ± 1.4

^1^ The °h values of hot-air-dried and freeze-dried *NP1648* flours were reduced to the first quadrant of the colour space.

**Table 3:** Proximal composition (moisture, fat, protein, carbohydrates, and ash content) for hot-air-dried and freeze-dried SP flours from the three varieties under study. For each column, different letters indicate significant differences (*p* < 0.05).

Drying Method	Variety	Moisture Content (%)	Fat (%)	Protein (%)	Carbohydrates (%)	Ash (%)
HAD	*Bonita*	3.0 ^a^ ± 0.1	0.5 ^a^ ± 0.1	4.9 ^a^ ± 0.0	88.0 ^a^ ± 0.2	3.7 ^b^ ± 0.0
FD		3.7 ^c^ ± 0.3	0.3 ^a^ ± 0.1	4.8 ^a^ ± 0.1	87.6 ^a^ ± 0.1	3.6 ^b^ ± 0.1
HAD	*Bellevue*	5.2 ^e^ ± 0.1	0.7 ^b^ ± 0.1	5.6 ^b^ ± 0.0	84.5 ^b^ ± 0.2	4.0 ^c^ ± 0.1
FD		4.6 ^d^ ± 0.2	0.9 ^b^ ± 0.1	5.4 ^b^ ± 0.1	85.1 ^b^ ± 0.3	4.0 ^c^ ± 0.1
HAD	*NP1648*	2.2 ^b^ ± 0.2	0.4 ^a^ ± 0.1	6.2 ^c^ ± 0.0	87.9 ^a^ ± 0.3	3.3 ^a^ ± 0.1
FD		2.8 ^a^ ± 0.1	0.3 ^a^ ± 0.1	6.0 ^c^ ± 0.0	87.9 ^a^ ± 0.1	3.3 ^a^ ± 0.0

**Table 4:** Composition of mineral elements (mg/kg) of hot-air-dried and freeze-dried SP flours from the three studied varieties. For each column, different letters indicate significant differences (*p* < 0.05).

Drying Method	Variety	Na	K	Ca	Mg	P	S
HAD	*Bonita*	16.5 ^a^ ± 1.7	220.6 ^a^ ± 20.0	16.6 ^a^ ± 1.2	15.8 ^c^ ± 1.2	30.4 ^b^ ± 2.5	14.4 ^a^ ± 1.0
FD		16.5 ^a^ ± 0.9	221.2 ^a^ ± 10.6	13.1 ^a^ ± 0.8	12.7 ^bc^ ± 0.6	28.6 ^b^ ± 1.2	13.7 ^a^ ± 0.6
HAD	*Bellevue*	7.1 ^b^ ± 0.1	263.9 ^c^ ± 4.6	15.5 ^a^ ± 0.3	10.5 ^a^ ± 0.2	22.8 ^b^ ± 0.4	14.2 ^a^ ± 0.3
FD		7.7 ^b^ ± 0.1	253.5 ^c^ ± 6.7	13.9 ^a^ ± 0.2	10.1 ^a^ ± 0.2	23.7 ^b^ ± 0.7	14.0 ^a^ ± 0.2
HAD	*NP1648*	18.1 ^a^ ± 1.2	168.8 ^b^ ± 10.3	28.6 ^b^ ± 1.6	13.4 ^bc^ ± 0.7	34.1 ^a^ ± 2.1	16.6 ^a^ ± 1.0
FD		18.2 ^a^ ± 2.2	150.7 ^b^± 16.0	26.7 ^b^ ± 3.0	12.1 ^bc^ ± 1.3	33.7 ^a^ ± 4.0	15.1 ^a^ ± 1.8

**Table 5:** Bioactive composition (TPC and AOx from FRAP and DPPH methods (hydrophilic—MeOH; lipid—DCM fractions), TCC, β-carotene content, and TAC) for fresh SP and hot-air-dried and freeze-dried flours from the three studied varieties. For each column, different letters indicate significant differences (*p* < 0.05).

Processing	Variety	TPC (mg GAE/100 g db)	TCC (mg β-Carotene/100 g db)	TAC (mg Cyd-3-glu/100 g db)	β-Carotene (mg/100 g db)	FRAP (mmol FeSO_4_(7 H_2_O)/100 g db)	DPPH MeOH (µmol TE/100 g db)	DPPH DCM (µmol TE/100 g db)
Raw material	*Bonita*	76.4 ^a^ ± 8.3	0.4 ^a^ ± 0.1	7.4 ^a^ ± 0.7	n.d	23.8 ^a^ ± 1.0	12,487.5 ^c^ ± 1863.4	3330.8 ^d^ ± 140.5
HAD		23.3 ^b^ ± 3.8	0.3 ^a^ ± 0.1	5.0 ^a^ ± 0.6	n.d	9.7 ^c^ ± 0.4	3415.3 ^b^ ± 162.0	1147.6 ^a^ ± 72.0
FD		55.6 ^a^ ± 2.7	0.3 ^a^ ± 0.1	6.6 ^a^ ± 0.7	n.d	22.8 ^a^ ± 0.7	8184.2 ^a^ ± 68.5	1648.2 ^ab^ ± 161.6
Raw material	*Bellevue*	173.2 ^c^ ± 23.2	49.3 ^d^ ± 2.0	14.9 ^c^ ± 2.3	27.2 ^d^ ± 4.3	66.9 ^e^ ± 4.2	43,461.0 ^e^ ± 1192.7	5172.4 ^e^ ± 171.9
HAD		61.6 ^a^ ± 1.8	33.5 ^b^ ± 1.0	4.9 ^a^ ± 1.4	12.7 ^b^ ± 0.2	24.9 ^a^ ± 0.9	8414.1 ^a^ ± 47.9	3149.2 ^cd^ ± 308.1
FD		74.8 ^a^ ± 7.7	43.6 ^c^ ± 1.6	11.2 ^b^ ± 0.9	20.0 ^c^ ± 2.7	30.7 ^b^ ± 0.4	8123.6 ^a^ ± 227.0	2617.5 ^bcd^ ± 233.4
Raw material	*NP1648*	226.9 ^d^ ± 5.0	1.7 ^a^ ± 0.2	27.3 ^f^ ± 0.7	n.d	103.3 ^f^ ± 1.5	34,359.3 ^d^ ± 159.2	3106.3 ^cd^ ± 193.0
HAD		57.5 ^a^ ± 3.0	0.8 ^a^ ± 0.1	21.9 ^e^ ± 1.0	n.d	30.1 ^b^ ± 0.5	8164.3 ^a^ ± 22.7	1751.8 ^ab^ ± 128.3
FD		74.3 ^a^ ± 1.7	1.1 ^a^ ± 0.0	18.5 ^d^ ± 0.3	n.d	40.3 ^d^ ± 0.3	7853.1 ^a^ ± 88.0	2122.2 ^abc^ ± 158.7

n.d: non-detectable.

## Data Availability

Data will be made available upon reasonable request to the corresponding author.

## Supplementary Materials

The following supporting information can be downloaded at https://www.mdpi.com/article/10.3390/molecules29235771/s1, Table S1: Factor loading on the two principal components of each variable; Figure S1: Hierarchical cluster analysis dendrogram of the data matrix.
