# Supplementary material for: Gluten-Free Sweet Potato Flour: Effect of Drying Method and Variety on the Quality and Bioactivity
Source: Molecules. 2024 Dec 6;29(23):5771. doi: 10.3390/molecules29235771 (PMC11643455; doi:10.3390/molecules29235771)
Supplement: Supplementary file 1 [file molecules-29-05771-s001.zip › molecules-3331105-supplementary.pdf]

molecules

MDPI

Article

Gluten-Free Sweet Potato Flour: Effect of Drying Method and Variety on the Quality and Bioactivity

Nelson Pereira [1,2], Ana Cristina Ramos [1,3], Marco Alves [4], Vítor D. Alves [2,5], Cristina Roseiro [1,3], Manuela Vida [1], Margarida Moldão [2,5] and Marta Abreu [1,2,5,*]

[1] Unidade de Tecnologia e Inovação, INIAV—Instituto Nacional de Investigação Agrária e Veterinária, 2780-157 Oeiras, Portugal; isa128286@isa.ulisboa.pt (N.P.); cristina.ramos@iniav.pt (A.C.R.); cristina.roseiro@iniav.pt (C.R.); manuela.vida@iniav.pt (M.V.)
[2] LEAF—Linking Landscape, Environment, Agriculture and Food Research Center, Instituto Superior de Agronomia, Universidade de Lisboa, 1349-017 Lisboa, Portugal; vitoralves@isa.utl.pt (V.D.A.); mmoldao@isa.utl.pt (M.M.)
[3] GeoBioTec—Geobiociências, Geoengenharias e Geotecnologias, NOVA School of Science and Technology, Universidade Nova de Lisboa, 2829-516 Caparica, Portugal
[4] INOV.LINEA/TAGUSVALLEY—Science and Technology Park, 2200-062 Abrantes, Portugal; marco_alves@tagusvalley.pt
[5] Associate Laboratory TERRA, Instituto Superior de Agronomia, Universidade de Lisboa, 1349-017 Lisboa, Portugal
* Correspondence: marta.abreu@iniav.pt

Supplementary Material

Tables

Supplementary Material Table S1. Factor loading on the two principal components of each variable

Variable	PC1	PC2
MC	-0.92*	0.26
TPC	-0.48	-0.79*
DPPH MeOH	-0.44	-0.74*
DPPH DCM	-0.80*	-0.32
FRAP	-0.15	-0.94*
TCC	-0.97*	-0.01
β-carotene	-0.95*	-0.00
TAC	0.37	-0.86*
Prot	-0.02	-0.80*
Fat	-0.89*	0.13
Ash	-0.83*	0.49
Carbo	0.98*	-0.04

* marked loading are > 0.70.

Figures

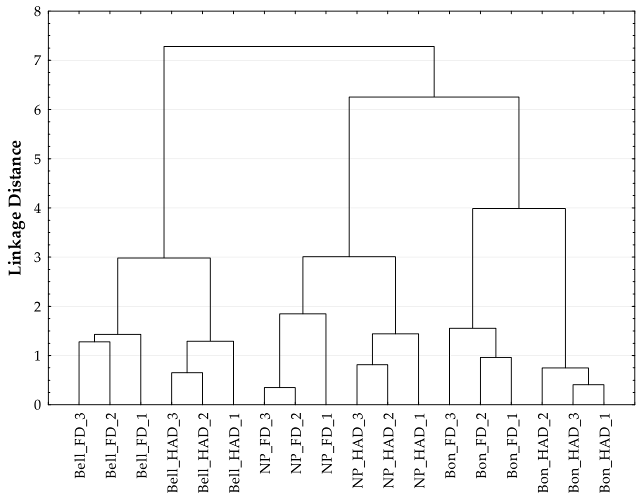

Supplementary Material Figure S1. Hierarchical cluster analysis dendrogram of the data matrix.
